# Interfacial Reactions between Sn-Based Solders and n-Type Bi_2_(Te,Se)_3_ Thermoelectric Material

**DOI:** 10.3390/ma17092158

**Published:** 2024-05-05

**Authors:** Chao-Hong Wang, Chun-Wei Chiu, Mei-Hau Li

**Affiliations:** Department of Chemical Engineering, National Chung Cheng University, Chiayi 621301, Taiwanshadow92022tw@yahoo.com.tw (M.-H.L.)

**Keywords:** thermoelectric materials, lead-free solders, interfacial reactions, Bi_2_(Te,Se)_3_

## Abstract

This study investigated the interfacial reactions between n-type Bi_2_(Te,Se)_3_ thermoelectric material, characterized by a highly-oriented (110) plane, and pure Sn and Sn-3.0Ag-0.5Cu (wt.%) solders, respectively. At 250 °C, the liquid-state Sn/Bi_2_(Te,Se)_3_ reactions resulted in the formation of both SnTe and BiTe phases, with Bi-rich particles dispersed within the SnTe phase. The growth of the SnTe phase exhibited diffusion-controlled parabolic behavior over time. In contrast, the growth rate was considerably slower compared to that observed with p-type (Bi,Sb)_2_Te_3_. Solid-state Sn/Bi_2_(Te,Se)_3_ reactions conducted between 160 °C and 200 °C exhibited similar interfacial microstructures. The SnTe phase remained the primary reaction product, embedded with tiny Bi-rich particles, revealing a diffusion-controlled growth. However, the BiTe layer had no significant growth. Further investigation into growth kinetics of intermetallic compounds and microstructural evolution was conducted to elucidate the reaction mechanism. The slower growth rates in Bi_2_(Te,Se)_3_, compared to the reactions with (Bi,Sb)_2_Te_3_, could be attributed to the strong suppression effect of Se on SnTe growth. Additionally, the interfacial reactions of Bi_2_(Te,Se)_3_ with Sn-3.0Ag-0.5Cu were also examined, showing similar growth behavior to those observed with Sn solder. Notably, compared with Ag, Cu tends to diffuse towards the interfacial reaction phases, resulting in a high Cu solubility within the SnTe phase.

## 1. Introduction

Thermoelectric (TE) modules have a variety of applications, serving not only as coolers or refrigerators but also as power generators by recovering waste heat, thereby enhancing energy usage efficiency. Over the past decade, concerns regarding global warming and green energy issues have gained increasing prominence, driving greater attention and research into the development of advanced thermoelectric materials with improved figure of merit [1,2,3]. Bi_2_Te_3_-based alloys, including p-type (Bi,Sb)_2_Te_3_ and n-type Bi_2_(Te,Se)_3_, are extensively utilized in commercial thermoelectric devices due to their superior TE performance across the low-temperature range from room temperature up to approximately 550 K [4,5].

In a typical thermoelectric device, numerous p- and n-type TE pillars are arranged electrically in series but thermally in parallel. These pillars are interconnected by soldering with Cu electrodes positioned between two ceramic substrates [2]. The solder paste is applied to Cu electrodes and reflowed with TE pillars. Sn-based lead-free solders, such as Sn-Ag-Cu (SAC) and Sn-Bi eutectic solders, are commonly used in commercial TE devices [6,7,8]. To prevent the interfacial reactions between solder and TE materials, a thin diffusion barrier, such as electrodeposited Ni, is introduced onto the soldering plane of TE materials. In recent years, extensive investigations have been conducted on the correlated interfacial reactions of lead-free solders/diffusion barriers/TE materials [9,10,11,12,13,14,15]. Electroless nickel/immersion gold (ENIG) and electroless nickel/electroless palladium/immersion gold (ENEPIG) are commonly used surface finishing processes for the diffusion barrier. In addition to Ni, Co is also frequently studied and recommended as a promising diffusion barrier material because it provides a stable interface [16,17,18].

The diffusion barrier layer plays a critical role in preventing reactions between the solder and the TE substrate, even though it is typically thin. Once it is consumed and depleted due to reactions with the solder, the solder can continue to diffuse and react with the TE substrate. Understanding the fundamental interfacial reactions between TE materials and solders is essential for assessing the reliability of solder joints in TE modules [19,20,21,22,23,24]. The prior study [23] on the interfacial reactions between p-type (Bi,Sb)_2_Te_3_ and Sn at 180 °C revealed rapid formation of both SnTe and SnSb, with reaction-limited linear growth rates with ~15 μm/h and 4.3 μm/h, respectively. As switching to SAC solder, the growth of intermetallic compounds (IMCs) was notably suppressed, attributed to the thin Ag-rich phase layer accumulated between SnTe and (Bi,Sb)_2_Te_3_. In the liquid-state Sn/(Bi,Sb)_2_Te_3_ reactions at 250 °C [24], the overall IMC growth rates were unusually rapid, involving three regions: the porous SnTe, the alternating structure of SnTe and solder, and the SnTe/Sn_3_Sb_2_ alternating layer.

However, until now, there has been no systematic study on the interfacial reactions between solder and n-type Bi_2_(Te,Se)_3_. In this study, we examined the interfacial reactions of Bi_2_(Te,Se)_3_ with Sn and SAC 305 solders. The liquid-state and solid-state reactions were carried out at 250 °C and 160–200 °C, respectively. In addition to investigating the interfacial microstructures, the kinetics of IMC growth were also studied. It was observed that the reaction behaviors with n-type Bi_2_(Te,Se)_3_ were different from those of p-type (Bi,Sb)_2_Te_3_. We propose a correlated reaction mechanism for further discussion.

## 2. Materials and Methods

The interfacial reactions of n-type Bi_2_(Te,Se)_3_ substrates with both pure Sn and Sn-Ag-Cu solders, were studied. The commercial n-type Bi_2_(Te,Se)_3_ substrates with a size of 4 × 4 × 2 mm^3^ were purchased from Kryotherm and fabricated using the zone-melting method. The SAC305 solder, with a total weight of 2 g, was prepared from its constituent pure elements (99.99%). They were weighed using an analytical balance with an accuracy of 0.1 mg, based on weight percentage. Subsequently, they were encapsulated in a quartz tube under a vacuum of below 10^−2^ torr. The SAC305 solder was homogenized in a furnace at 800 °C for 1 day and then quenched in water. The mass loss of the SAC305 alloy was confirmed to be below 0.1% after homogenization. The Sn or SAC305 ingot was sliced into several 2-mm-thick discs. The Bi_2_(Te,Se)_3_ substrates and the solder discs were polished and then cleaned with deionized water. Subsequently, the Bi_2_(Te,Se)_3_ substrates were rinsed with a rosin mildly activated (RMA) flux.

During liquid-state interfacial reactions, the solder disc was placed on the Bi_2_(Te,Se)_3_ substrate, and the sample was heated on a hot plate at 250 °C for a specific period, ranging from 1 min to 30 min. Upon completion of the reaction, the reaction couple was immediately quenched with water. For the solid-state interfacial reactions, the reaction couples were solder together on a hot plate at 250 °C. Once the molten solder got wetted with the Bi_2_(Te,Se)_3_ substrate, the reaction couples were promptly quenched. The solid-state reactions were conducted at temperatures of 160 °C, 180 °C, and 200 °C for different durations. After heat-treatment, the reaction couples were sectioned, mounted in epoxy resin, and subsequently subjected to metallographic grinding and polishing.

The reaction couples were immersed in a Sn-etching solution (CH_3_OH + 2% HCl + 5% HNO_3_) for 2~3 sec to slightly etch the Sn solder matrix, revealing the distinct interfacial microstructure. Additionally, to further observe the morphology of the reaction phases, the sample was conducted by deep-etching. Similarly, the reaction couple was immersed in a Sn-etching solution for several minutes to completely remove the solder, revealing the grains of reaction phases. The interfacial results were characterized using field-emission scanning electron microscopy (FESEM, FEI Quanta 3D FEG) with back-scattered electron image (BEI) mode. For the deep-etching samples, the grain morphologies of the reaction products were observed using secondary electron image (SEI) mode. The compositions of the reaction phases were determined using field emission electron probe microanalyzer (EPMA, JXA-8530F, JEOL Ltd., Tokyo, Japan), equipped with four wavelength-dispersive spectrometers (WDS), capable of detecting elements ranging from B(5) to U(92). The Bi_2_(Te,Se)_3_ substrate underwent X-ray diffraction (XRD, Bruker D8) analysis with Cu-K_α_ radiation (λ = 1.54056 Å). The XRD patterns were compared with the data in the Joint Committee on Powder Diffraction Standards (JCPDS) database. To analyze the growth kinetics of IMCs, the thickness of the IMC layer was calculated by dividing the area of the reaction phase by the length of the interface. In addition, multiple measurements were conducted at different locations along the interface to ensure accuracy and reliability.

## 3. Results and Discussion

### 3.1. Liquid-State Sn/n-Type Bi_2_(Te,Se)_3_ Interfacial Reactions

**Figure 1:**
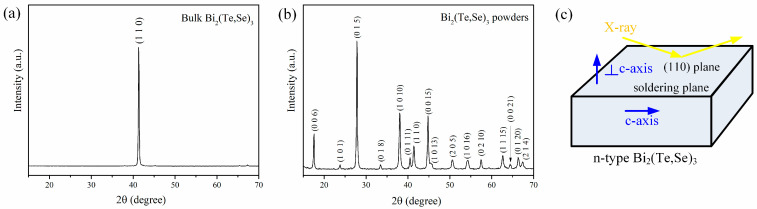
XRD spectra of n-type Bi_2_(Te,Se)_3_ thermoelectric material: (**a**) the commercial bulk material, (**b**) ground Bi_2_(Te,Se)_3_ powders, and (**c**) schematically illustrating the Bi_2_(Te,Se)_3_ substrate with a preferred orientation of (1 1 0).

The composition of the commercial n-type Bi_2_(Te,Se)_3_ thermoelectric material was measured using EPMA to be 40.1 at.%Bi-6.0 at.%Se-53.9 at.%Te. The composition is consistent with the typical commercial n-type TE material, and can be expressed as Bi_2_(Te_0.9_Se_0.1_)_3_. It was further characterized through XRD analysis, as shown in Figure 1a, obtained from the soldering plane. The XRD findings revealed a strong orientation of the n-type TE substrate, evidenced by a single pronounced diffraction peak at *2*-theta of 41.2°, corresponding to the (110) plane of Bi_2_(Te,Se)_3_. This compound exhibits a typical rhombohedral layered-structure along the c-axis (JCPDS # 50-0954, space group: R-3m with lattice parameters *a* = 0.4374 nm and *c* = 3.0424 nm). Moreover, the bulk Bi_2_(Te,Se)_3_ material was grounded to powders for further analysis. Figure 1b demonstrates that complex diffraction peaks closely match those of the JCPDS standard patterns, indicating the poly-crystalline nature of the material. Notably, the prominent peak at 27.8° corresponds to the (015) plane. According to the XRD results, the soldering plane is associated with the (110) crystalline plane, indicating alignment with the c-axis, as illustrated in Figure 1c.

**Figure 2:**
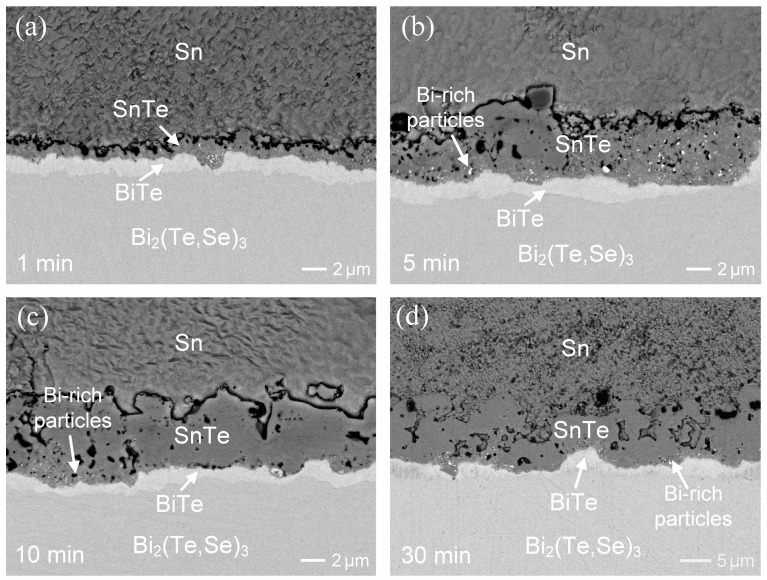
BEI micrographs of the Sn/Bi_2_(Te,Se)_3_ reactions at 250 °C for (**a**) 1 min, (**b**) 5 min, (**c**) 10 min, and (**d**) 30 min.

The interfacial reactions between the liquid-state Sn solder and Bi_2_(Te,Se)_3_ at 250 °C for various durations are displayed in Figure 2a–c. After 1 min of reaction, as shown in Figure 2a, two distinct reaction layers with dense structures were observed at the interface. A bright phase layer, approximately 1.2 µm thick, was located at the side of the Bi_2_(Te,Se)_3_ substrate, while a dark layer, around 1.1 µm thick, was formed adjacent to the solder. With an increase in reaction time to 3 min, both the reaction layers slightly thickened, although the interfacial microstructure remined similar.

Furthermore, the reaction was prolonged to 5 min, as shown in Figure 2b. EPMA analysis revealed the composition of the dark phase as 53.1 at.%Sn-0.2 at.%Bi-3.2 at.%Se-43.5 at.%Te, which was identified as the SnTe phase. Based on the phase equilibria of the Sn-Bi-Te system and the Se-Sn-Te system [25,26,27], the solubilities of Bi and Se in the SnTe phase at 250 °C is about 10 at.%Bi and 4at.%Se, respectively, consistent with the observed composition of SnTe in the interfacial reactions. The bright phase contained 4.1 at.%Sn-49.7 at.%Bi-3.8 at.%Se-42.4 at.%Te, suggesting that it was the BiTe phase with ~4 at.%Sn and ~4 at.%Se solubilities. The Sn and Se atoms could substitute the Te lattice sites in the BiTe phase. However, there is no available data in the literature regarding the composition or solubilities of Sn and Se in the BiTe phase. The solder region near the interface exhibited a solubility of 0.5 at.%Bi, 0.1 at.%Se, and 2.3 at.%Te. Notably, some tiny bright particles were dispersed in the SnTe phase.

Figure 2c shows the interfacial results after reacting for 10 min. The dark SnTe layer, measuring approximately 4.5 µm in thickness, appeared notably thicker than the bright BiTe layer, which was approximately 1.6 thick. Evidently, the SnTe phase was composed of large particulates with presence of voids. Similarly, small bright particles were observed within SnTe phase near the BiTe layer. Based on the contrast, these particles were suggested to be Bi precipitates, likely formed due to an oversaturation of Bi. Upon increasing the reaction time to 30 min (Figure 2d), in addition to the growth of IMC layers, the interfacial microstructure remained stable with no significant evolution.

**Figure 3:**
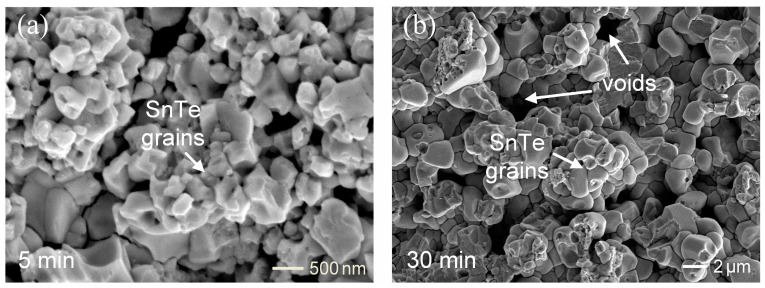
The grain morphologies of the SnTe phase in the Sn/Bi_2_(Te,Se)_3_ reactions at 250 °C for (**a**) 5 min and (**b**) 30 min.

To further explore the morphology of SnTe phase, the Sn solder of the reaction couple was removed through deep etching. Figure 3a,b display the top-view SEI micrographs showing displaying the grain morphologies of the SnTe phase after 5 min and 30 min of reaction, respectively. The SnTe phase exhibits round grains with numerous voids. In the 5-min sample, the grain size was less than 500 nm. In contrast, the grain size increased to approximately 2 µm in the 30-min sample. This indicated that the grain coarsening occurred with aging time during the soldering reaction. From a series of microstructural observations, the SnTe phase, composed of fine grains embedded with Bi-rich particles, exhibited grain coarsening and void formation. This void formation was attributed to Bi dissolution into the solder, resulting in voids within the SnTe phase.

Moreover, it was noted that the fraction of Bi-rich particles in the SnTe layer at 10 min was significantly lower than those at 1 and 5 min. As the reaction proceeded, the SnTe grains gradually coarsened and became denser, resulting in a reduction in voids. Additionally, the Bi-rich particles dissolved into the molten solder, leading to a decrease in the amounts of Bi-rich particles in the SnTe phase. Similar phenomena were also observed after a 30-min reaction. In the n-type Bi_2_(Te,Se)_3_, Se substitutes Te in the solid solution of the Bi_2_(Te,Se)_3_ lattice. Analysis of the reaction products of SnTe and BiTe revealed the Se concentrations of approximately 3~4 at.%. This observation suggests that Se could also substitute Te in the lattices of SnTe or BiTe. The low Se concentration in the solder near the interface indicated a very limited solubility of Se in liquid Sn.

**Figure 4:**
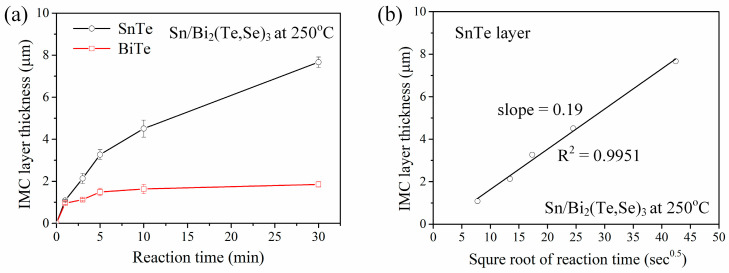
IMC growth kinetics analysis of the liquid-state Sn/Bi_2_(Te,Se)_3_ reactions at 250 °C. (**a**) Average thicknesses of SnTe and BiTe layers as a function of aging time. (**b**) The plot of average thickness of SnTe versus the square root of reaction time.

Figure 4a show the average thicknesses of both the SnTe and BiTe layers plotted against aging time at 250 °C. The SnTe layer exhibited a continuous increase in thickness with aging time. In contrast, the growth of the BiTe layer gradually ceased after 5 min of reaction and maintained a thickness of ~1.6 µm. The IMC growth can be described by the following equation [28]:

(1) $$x=k\;\times \;t^{n}$$

where *x* represents the IMC thickness, *k* is the rate constant, *t* is the aging time, *n* is the time exponent. In general, the IMC growth is controlled by lattice diffusion, following a parabolic relationship with aging time, where the expected value of *n* is 0.5. In Figure 4a, the SnTe phase exhibited a nearly parabolic growth. The natural logarithm plot of SnTe thickness versus aging time yielded an n value of 0.58, close to 0.5. Accordingly, the growth was assumed to be diffusion-controlled and the average thickness of SnTe plotted against the square root of reaction time, as shown in Figure 4b. The data fitted well to a linear trend. From the slope, the diffusion-controlled growth rate constant, *k_diff_*, for the SnTe phase was determined to be 0.19 µm/s^0.5^. The diffusion-controlled growth rate constant is crucial in interfacial reactions, as it significantly influences the reaction kinetics and resulting microstructure. Understanding this constant helps elucidate the reaction mechanism, predict IMC growth behavior, and even determine diffusion coefficients.

During the initial soldering process at the Sn/Bi_2_(Te,Se)_3_ interface, the Te component of Bi_2_(Te,Se)_3_ substrate reacted with the solder, leading to the formation of the SnTe phase. Simultaneously, a portion of Bi from the substrate dissolved into the solder. The remaining Bi and Te constituents of the Bi_2_(Te,Se)_3_ substrate subsequently transformed to the stable BiTe phase. As the reaction progressed, the primary diffusion species, Sn, continued to diffuse to reacted with the Te, which diffused across the BiTe phase from the substrate, resulting in the additional SnTe formation. The accumulated oversaturated Bi precipitated as Bi-rich particles within the SnTe phase.

In the previous study [24] on the Sn/p-type (Bi,Sb)_2_Te_3_ reactions at 250 °C, the observed reaction phases consisted of three zones: the porous SnTe, the alternating-layer microstructure of SnTe and liquid solder, and the SnTe/Sn_3_Sb_2_ alternating layer. The reaction-limited linear growth exhibited an exceptionally rapid rate, approximately 15 μm/min. The reaction phase zone exceeded 450 μm after 30 min of reaction. However, in the present study, the reactions of Bi_2_(Te,Se)_3_ with Sn demonstrated that the growth of SnTe and BiTe IMCs was relatively sluggish. The overall IMC thickness reached merely ~10 μm after 30 min of aging. These finding suggested that the soldering reactions of n-type Bi_2_(Te,Se)_3_ were considerably slower compared to those of p-type (Bi,Sb)_2_Te_3_.

### 3.2. Solid-State Sn/n-Type Bi_2_(Te,Se)_3_ Interfacial Reactions

**Figure 5:**
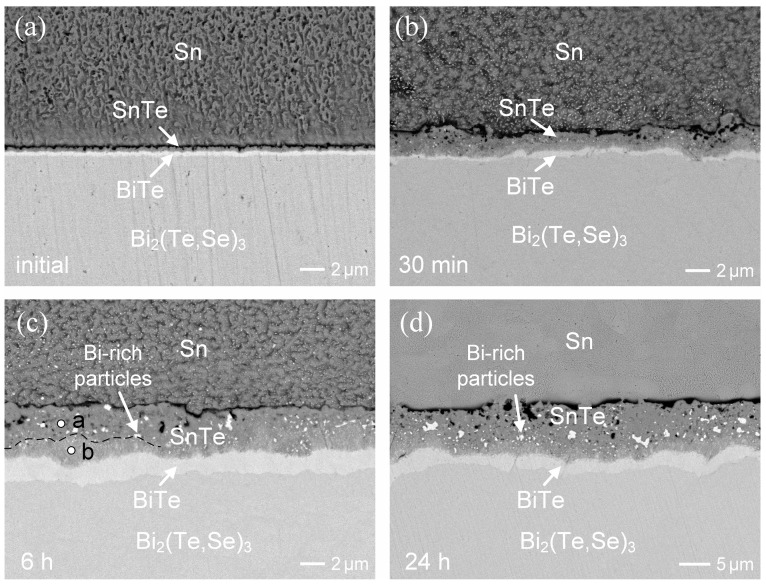
BEI micrographs of the Sn/Bi_2_(Te,Se)_3_ reactions at 180 °C for different durations: (**a**) the initial interface, (**b**) 30 min, (**c**) 6 h, and (**d**) 24 h.

The initial interface of the as-soldered Sn/Bi_2_(Te,Se)_3_ couple is shown in Figure 5a. The overall reaction layer was less than 1 µm, but two distinct reaction phases were clearly visible, similar to those observed in the liquid-state interfacial reactions. Figure 5b–d present the interfacial results at 180 °C for 30 min, 6 h, and 24 h, respectively. After 30 min of aging, the overall reaction phases measured ~2 µm, with the dark reaction layer appearing relatively thicker than the bright one. As the aging time increased, the reaction phase gradually thickened. In Figure 5c, the compositions of the dark phase showed slight variations: 51.2 at.%Sn-1.9 at.%Bi-2.9 at.%Se-44.0 at.%Te (near the solder side, point a) and 40.3 at%Sn-14.9 at.% Bi-3.7 at.%Se-41.1 at.%Te (near the Bi_2_(Te,Se)_3_ side, point b). Based on the correlated phase diagrams [25,26], these compositions are consistent with the SnTe phase, although the inner SnTe phase demonstrated a higher Bi solubility.

Additionally, the bright phase was identified as 3.7 at.%Sn-53.1 at.%Bi-3.2 at.%Se-40.0 at.%Te, corresponding to the BiTe phase with minor solubility of Sn and Se. Notably, there were numerous dispersed bright particles within the SnTe phase. The EPMA analysis revealed that they contained a very high Bi content, suggesting that they were the Bi-rich particles.

**Figure 6:**
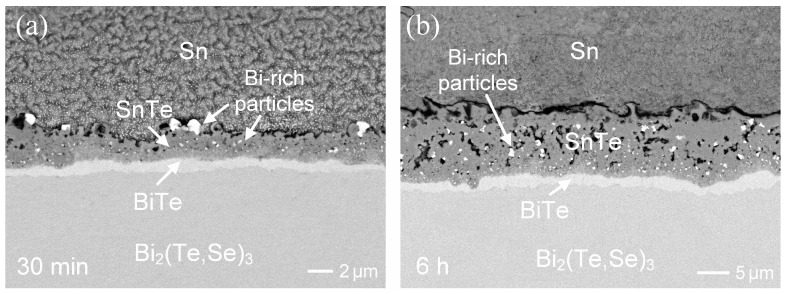
BEI micrographs showing the Sn/Bi_2_(Te,Se)_3_ reactions at 200 °C for (**a**) 30 min and (**b**) 6 h.

Similar interfacial microstructures were observed in the reaction after 24 h. The dark SnTe phase was ~11 µm, while the bright BiTe phase was ~2.4 µm. The presence of numerous tiny Bi-rich particles in the SnTe phase could be attributed to their precipitation due to oversaturation. The aging temperature was raised to 200 °C. The interfacial reaction behaviors showed no significant difference, but the IMC growth rate greatly accelerated. As shown in Figure 6a, both the SnTe and BiTe layers were formed, with a combined thickness of ~3.4 µm after only 30 min of reaction. After 6 h of aging (Figure 6b), the SnTe layer reached a thickness of ~10 µm, embedded with numerous Bi-rich particles, and exhibited a loose-structure with voids.

It is intriguing that a significant number of voids were observed in the SnTe phase during the solid-state interfacial reactions. This is likely attributed to liquation occurring during the solid-state reaction [29]. With the eutectic temperature of Sn-Bi at only 139 °C, and the Bi saturation concentration in Sn being approximately 10 at.% at 160 °C and 4 at.% at 200 °C, the solder enters a two-phase equilibrium of Sn and liquid as Bi is oversaturated in Sn. As Bi diffuses and dissolves into the Sn solder, exceeding the saturated concentration, local liquation in the interfacial zone could occur in the solid-state reactions. Consequently, the local liquation could cause the solder to fill the voids within SnTe grains, resulting in a loosely structured SnTe. During sample etching, the solder was removed, revealing these voids.

**Figure 7:**
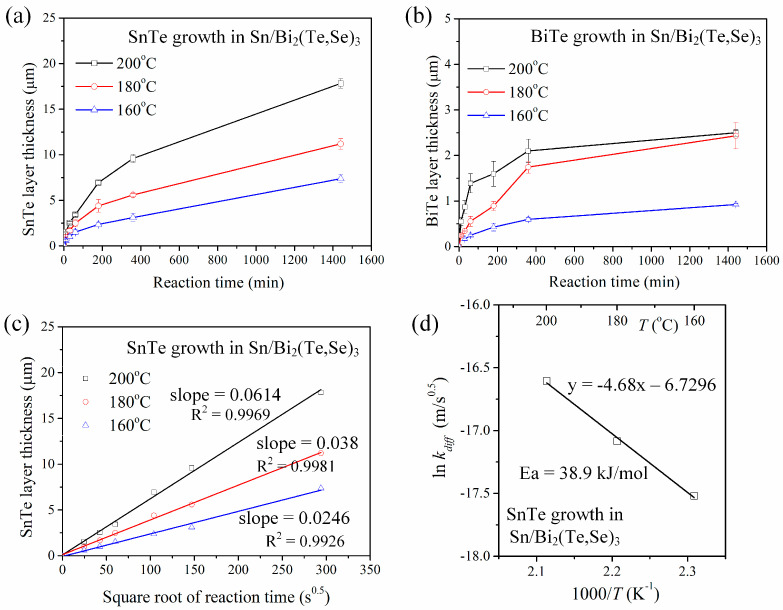
IMC growth kinetics analysis of the solid-state Sn/Bi_2_(Te,Se)_3_ reactions at 160 °C, 180 °C, and 200 °C. (**a**) Average thicknesses of SnTe layers as a function of aging time. (**b**) Average thicknesses of BiTe layers as a function of aging time. (**c**) The plot of average thickness of SnTe versus the square root of aging time. (**d**) Arrhenius plot of ln *k_diff_* versus 1000/*T* for the SnTe growth.

To further investigate the IMC growth kinetics, the interfacial reactions were also conducted at 160 °C. Figure 7a,b show the average thicknesses of the SnTe and BiTe layers, respectively, as a function of aging time at 160 °C, 180 °C, and 200 °C. In the natural logarithm plot of SnTe thickness versus aging time, the *n* values were determined to be 0.49, 0.49, and 0.51 for the reactions at 160 °C, 180 °C, and 200 °C, respectively. These *n* values were very close to 0.5, indicating that the SnTe growth was govern by diffusion and followed a parabolic relationship with aging time. Assuming the growth model with *n* = 0.5, the plot of the average thickness of SnTe versus the square root of aging time is depicted in Figure 7c. The data showed a good fit of linear regression, with *R*^2^ values (the coefficient of determination) exceeding 0.99. The diffusion-controlled growth rate constants, *k_diff_*, can be determined from the linear-fitting slopes. The *k_diff_* values were 0.0614, 0.038, and 0.0246 µm/s^0.5^ at 200 °C, 180 °C, and 160 °C, respectively.

The growth rate constants increased with increasing aging temperature. To analyze the temperature dependent of the growth rate constants, it can be described by the Arrhenius equation:

(2) $$k=k_{0}\exp(-Q/RT)$$

where *k*_0_ is the temperature-independent pre-exponential factor, *Q* is the activation energy, *R* is the universal gas constant, and *T* is the absolute temperature. The Arrhenius plot presented in Figure 7d indicated that the activation energy was 38.9 kJ/mol and the pre-exponential factor, *k*_0_, was 0.0012 m/s^0.5^. These kinetics parameters can help predict the growth rate constants at different temperatures. For instance, the growth rate constant at 120 °C was estimated to be 0.0081 µm/s^0.5^. In comparison, in the Sn/p-type (Bi,Sb)_2_Te_3_ reaction, the SnTe growth was rapid and linear with aging time, suggested that it was reaction-controlled. The activation energy was quite high, 151.6 kJ/mol. Accordingly, the n-type Bi_2_(Te,Se)_3_ reaction was relatively lower, indicating that the SnTe growth is less sensitive to temperature changes.

**Figure 8:**
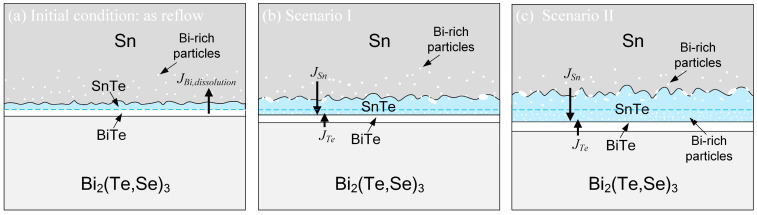
Schematic illustration of the solid-state Sn/Bi_2_(Te,Se)_3_ reactions: (**a**) the initial condition, (**b**) Scenario I, and (**c**) Scenario II.

Figure 8a–c schematically illustrate the reaction process of the solid-state Sn/Bi_2_(Te,Se)_3_ reactions. In Figure 8a, both the thin SnTe and BiTe layers are initially formed during the short-time soldering. The Bi from the Bi_2_(Te,Se)_3_ substrate is dissolved into the solder and precipitates as tiny Bi particles in the vicinity of the interface. As shown in Figure 8b, the dominant diffusion species of Sn diffuses and reacts with the Te atoms, which diffuses across the thin BiTe layer from the Bi_2_(Te,Se)_3_ substrate, resulting in the formation of the SnTe phase. The BiTe layer is passively formed and hinders the Sn atoms, thus acting as a diffusion barrier for the Te atoms. Additionally, the dispersed Bi particles in the solder gradually grow in size. With the reaction progressing, the SnTe layer continued to grow, while the BiTe layer maintain a thickness of ~2.5 µm at 180 °C and 200 °C after 360 h. In certain local regions, the BiTe phase appeared to become thinner in the later stages of the reaction, suggesting the occurrence of BiTe decomposition. As illustrated in Figure 8c, the diffusing Sn atoms could react with the BiTe phase, leading to the decomposition of the BiTe layer to form the SnTe phase and oversaturated Bi particles. Consequently, the SnTe phase exhibits two distinct microstructures, with a significant number of small Bi-rich particles embedded within the region near the BiTe layer.

**Figure 9:**
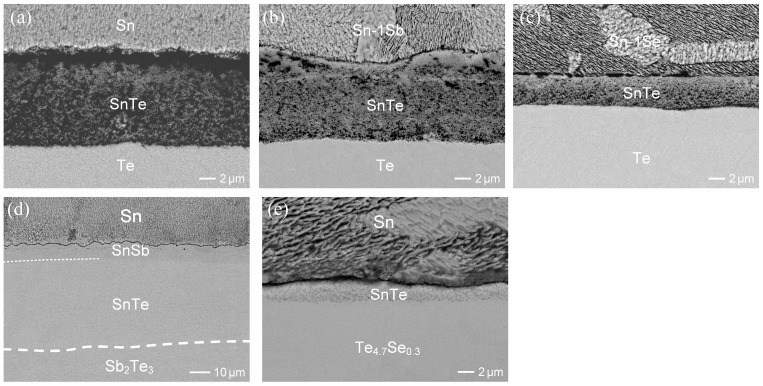
BEI micrographs showing the reactions of solders with various substrates at 180 °C for 6 h: (**a**) Sn/Te, (**b**) Sn-1wt.%Sb/Te, (**c**) Sn-1wt.%Se/Te, (**d**) Sn/Sb_2_Te_3_, and (**e**) Sn/Te_4.7_Se_0.3_. In Figure (**d**), the dashed lines represent the boundaries between SnSb and SnTe, and between SnTe and Sb_2_Te_3_, respectively.

In the Sn/p-type (Bi,Sb)_2_Te_3_ reactions, both the SnTe and SnSb phases were fast formed with linear growth rates of 15.1 μm/h and 4.3 μm/h, respectively, at 180 °C [23]. In comparison, the growth of IMCs in the reactions involving n-type Bi_2_(Te,Se)_3_ was significantly suppressed. To further examine the influence of Se and Sb additives on the IMC growth, the interfacial reactions of Te with Sn, Sn-1wt.%Sb, and Sn-1wt.%Se were conducted at 180 °C for 6 h, as shown in Figure 9a–c. The thicknesses of the formed SnTe phases were ~12 µm, ~12 µm, and ~4 µm, respectively. It is evident that the Se additive in the solder inhibits IMC growth, while the Sb additive shows no significant influence. Moreover, the reactions of Sn with Sb_2_Te_3_ and Te_4.7_Se_0.3_ were also performed for comparison, as shown in Figure 9d,e. The overall thicknesses of SnTe and SnSb layers in the Sb_2_Te_3_ reaction was approximately 55 μm. In contrast, the SnTe phase formed in the Te_4.7_Se_0.3_ reaction was only around 2 μm. The results clearly indicated that the presence of the Se additive in the Te substrate would greatly inhibit the IMC growth, while it was notably enhanced with the Sb additive. As a result, there was a significant difference in IMC growth between reactions involving p-type (Bi,Sb)_2_Te_3_ and n-type Bi_2_(Te,Se)_3_ substrates. However, the underlying inhibiting mechanism of the Se additive remains unclear and requires further investigation.

### 3.3. SAC305/n-Type Bi_2_(Te,Se)_3_ Interfacial Reactions

**Figure 10:**
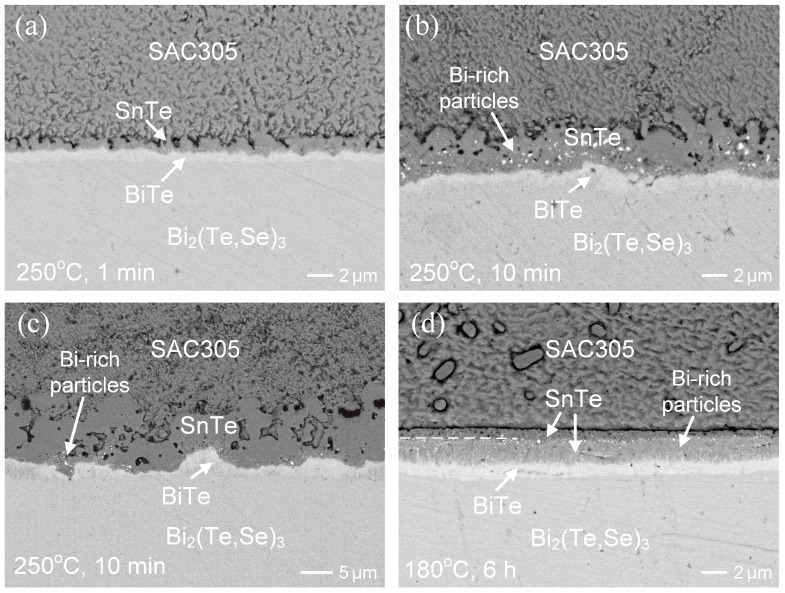
BEI micrographs of the SAC305/Bi_2_(Te,Se)_3_ reactions, (**a**–**c**) at 250 °C for 1 min, 10 min, and 30 min, respectively; (**d**) at 180 °C for 6 h.

**Figure 11:**
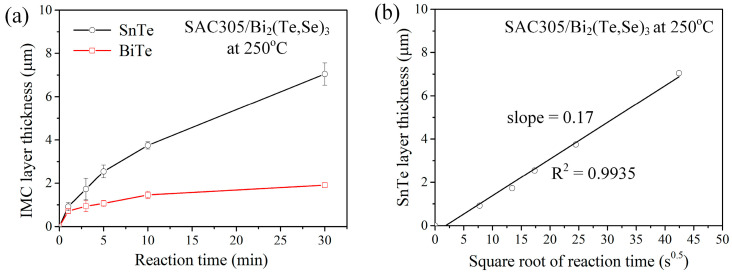
IMC growth analysis of the liquid-state SAC305/Bi_2_(Te,Se)_3_ reactions at 250 °C. (**a**) Average thicknesses of SnTe and BiTe layers as a function of aging time. (**b**) The plot of average thickness of SnTe versus the square root of reaction time.

The interfacial reactions of Bi_2_(Te,Se)_3_ substrate with SAC305 solder were conducted at 250 °C. In Figure 10a, after 1 min of reaction, similar to the Sn/Bi_2_(Te,Se)_3_ reactions, two reaction phases layers of SnTe and BiTe were observed. The dark SnTe phase, exhibiting a scallop morphology, was predominant. As the reaction time increased to 10 min and 30 min, as displayed in Figure 10b and Figure 10c, respectively, not only did the IMC layers grow thicker, but also the grains of SnTe gradually coarsened. Additionally, the Bi precipitates were also observed within the SnTe phase. In fact, Bi contained in the SnTe phase would gradually dissolve to the molten solder. Consequently, the number of Bi-rich particles in the 10-min case was much higher than that of the 30-min case. Figure 11a shows the growth of SnTe and BiTe layers over time. In the natural logarithm plot, the *n* value for the SnTe growth was 0.6, indicating that it was nearly diffusion-controlled. As illustrated in Figure 11b, the *k_diff_* was determined to be 0.17 μm/s^0.5^. This value was slightly lower compared to the reactions with Sn.

The SAC305/Bi_2_(Te,Se)_3_ reactions were further conducted at 180 °C for various durations ranging from 1 h to 24 h. The interfacial microstructures resembled those observed in the Sn/Bi_2_(Te,Se)_3_ reactions. Besides the gradual growth of IMCs, there was no significant evolution in the interfacial morphologies. Figure 10d illustrates the reaction at 180 °C for 6 h. Both the SnTe and BiTe phases were present at the interface. The bright BiTe phase was characterized by its composition: 1.3 at.%Sn-49.9 at.%Bi-3.5 at.%Se-44.3 at.%Te-0.4 at.%Ag-0.6 at.%Cu. The SnTe phase exhibited two distinct regions, with the one adjacent to the BiTe side containing numerous bright particles. Its composition contained 38.7 at.%Sn-15.1 at.%Bi-2.0 at.%Se-40.2 at.%Te-0.2 at.%Ag-3.8 at.%Cu, indicating that it was the SnTe phase mixed with tiny Bi-rich particles. Additionally, it was noted that the Cu content was higher than Ag, suggesting the facile diffusion and dissolution of Cu into the SnTe phase.

## 4. Conclusions

The interfacial reactions of n-type Bi_2_(Te,Se)_3_ substrate with Sn and SAC305 solders were carefully examined. The soldering plane of Bi_2_(Te,Se)_3_ substrate exhibited a highly-orientated (1 1 0) crystalline plane. In the liquid Sn/Bi_2_(Te,Se)_3_ reactions, both the SnTe and BiTe phase were formed simultaneously, and Bi particles precipitated within the SnTe phase. The growth of the SnTe phase was govern by diffusion and followed a slow growth rate constant of be 0.19 µm/s^0.5^. Nevertheless, the BiTe phase ceased to grow at the later stage of the reaction. In the solid-state reactions, similar interfacial microstructures were observed, where both the SnTe and BiTe phases were present. Notably, the presence of numerous embedded Bi particles in the SnTe phase near the BiTe layer was attributed to the reaction of Sn with the BiTe phase, leading to the decomposition and formation of SnTe and Bi particles. In the solid-state Sn/Bi_2_(Te,Se)_3_ reactions at temperatures ranging from 160 °C to 200 °C, the SnTe remained the primary reaction product, accompanied by the formation of the BiTe phase. Bi precipitates were dispersed within the SnTe phase near the BiTe phase. The growth of the SnTe phase revealed diffusion-controlled behavior. Additionally, the corresponding growth kinetics parameters were determined, and the mechanism of the reactions was illustrated and interpreted. The IMC growth rates in the reactions with n-type Bi_2_(Te,Se)_3_ were considerably slower than those involving p-type (Bi,Sb)_2_Te_3_. Based on a series of experimental tests, it was suggested that the presence of Se in the Bi_2_(Te,Se)_3_ significantly inhibits the growth of SnTe. The SAC305 solder was also used to conduct the reactions with Bi_2_(Te,Se)_3_. The interfacial microstructure and the IMC growth rate were found to be similar to those observed with pure Sn.

## Data Availability

The raw data supporting the conclusions of this article will be made available by the authors on request.
